# Recruiting a New Substrate for Triacylglycerol Synthesis in Plants: The Monoacylglycerol Acyltransferase Pathway

**DOI:** 10.1371/journal.pone.0035214

**Published:** 2012-04-16

**Authors:** James R. Petrie, Thomas Vanhercke, Pushkar Shrestha, Anna El Tahchy, Adam White, Xue-Rong Zhou, Qing Liu, Maged P. Mansour, Peter D. Nichols, Surinder P. Singh

**Affiliations:** 1 CSIRO Food Futures National Research Flagship, Canberra, ACT, Australia; 2 CSIRO Plant Industry, Canberra, ACT, Australia; 3 CSIRO Marine and Atmospheric Research, Hobart, TAS, Australia; Max Planck Institute for Chemical Ecology, Germany

## Abstract

**Background:**

Monoacylglycerol acyltransferases (MGATs) are predominantly associated with lipid absorption and resynthesis in the animal intestine where they catalyse the first step in the monoacylglycerol (MAG) pathway by acylating MAG to form diacylglycerol (DAG). Typical plant triacylglycerol (TAG) biosynthesis routes such as the Kennedy pathway do not include an MGAT step. Rather, DAG and TAG are synthesised *de novo* from glycerol-3-phosphate (G-3-P) by a series of three subsequent acylation reactions although a complex interplay with membrane lipids exists.

**Methodology/Principal Findings:**

We demonstrate that heterologous expression of a mouse MGAT acyltransferase in *Nicotiana benthamiana* significantly increases TAG accumulation in vegetative tissues despite the low levels of endogenous MAG substrate available. In addition, DAG produced by this acyltransferase can serve as a substrate for both native and coexpressed diacylglycerol acyltransferases (DGAT). Finally, we show that the *Arabidopsis thaliana* GPAT4 acyltransferase can produce MAG in *Saccharomyces cerevisiae* using oleoyl-CoA as the acyl-donor.

**Conclusions/Significance:**

This study demonstrates the concept of a new method of increasing oil content in vegetative tissues by using MAG as a substrate for TAG biosynthesis. Based on *in vitro* yeast assays and expression results in *N. benthamiana*, we propose that co-expression of a MAG synthesising enzyme such as *A. thaliana* GPAT4 and a MGAT or bifunctional M/DGAT can result in DAG and TAG synthesis from G-3-P via a route that is independent and complementary to the endogenous Kennedy pathway and other TAG synthesis routes.

## Introduction

**Figure 1:**
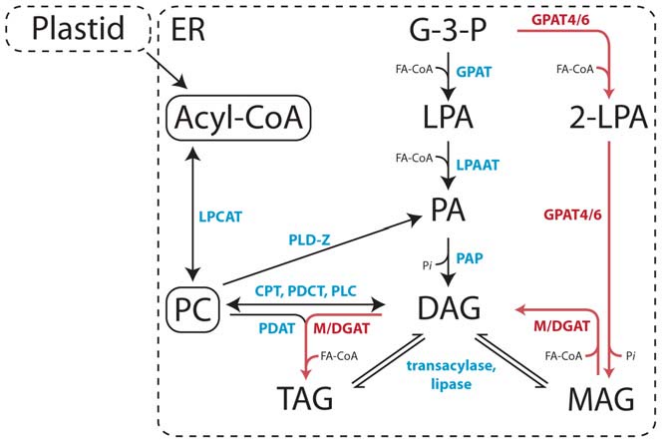
A simplified representation of general lipid metabolism in plant cells and the concept of the novel transgenic MGAT pathway proposed in this study. The substrates are G-3-P, glycerol-3-phosphate; LPA, lysophosphatidic acid; 2-LPA, *sn2*-lysophosphatidic acid; PA, phosphatidic acid; MAG, monoacylglycerol; DAG, diacylglycerol; TAG, triacylglycerol; PC, phosphatidylcholine. The enzymes are GPAT, glycerol-3-phosphate acyltransferase; GPAT4/6, denotes enzymes with both GPAT and phosphatase activity such as the *A. thaliana* GPAT4 and GPAT6; LPAAT, lysophosphatidic acid acyltransferase; PAP, phosphatidic acid phosphatase; MGAT, monoacylglycerol acyltransferase; DGAT, diacylglycerol acyltransferase; PDAT, phospholipid:diacylglycerol acyltransferase; CPT, CDP-choline:diacylglycerol cholinephosphotransferase; PDCT, phosphatidylcholine:diacylglycerol cholinephosphotransferase; PLC, phospholipase C; PLD-Z, phospholipase D Z-class; LPCAT, lysophosphatidylcholine acyltransferase; Pi, inorganic phosphate.

In higher eukaryotes the most direct biochemical pathway leading to triacylglycerol (TAG) biosynthesis is the acyl-CoA dependent Kennedy or glycerol phosphate pathway (Fig. 1) [1]. Diacylglycerol (DAG) is formed from glycerol-3-phosphate (G-3-P) by three subsequent acylation reactions. The initial acylation of G-3-P by glycerol-3-phosphate acyltransferase (GPAT) yields lysophosphatidic acid (LysoPA) which is further converted by lysophosphatidic acid acyltransferase (LPAAT) to phosphatidic acid (PA). Subsequent removal of a phosphate group from PA by the phosphatidate phosphatase (PAP) yields DAG, which is acylated by a diacylglycerol acyltransferase (DGAT) to produce TAG. Recent work, however, has demonstrated that TAG accumulation in plant cells is not necessarily as unidirectional as this traditional model indicates [2]–[3]. Rather complex interchanges occur between different neutral lipid pools of the Kennedy pathway and membrane lipids. Furthermore, there is a growing suite of enzymes known to interact with intermediates of Kennedy pathway (Fig. 1) thereby affecting neutral lipid production either directly or indirectly [4].

So far, efforts aimed at increasing TAG accumulation in plant tissues have largely focussed on altering the fatty acid biosynthesis and Kennedy pathways. Examples include the overexpression of single critical enzymes and the upregulation of multiple enzymatic steps by transcription factors (reviewed in [5]). One notable exception was the recent description of an *Arabidopsis thaliana* T-DNA mutant in which the homologue of a gene implicated in a human neutral lipid storage disease (*CGI-58*) was disrupted [6]. This resulted in the accumulation of neutral lipids in vegetative tissue in a manner similar to that observed in the human Chanarin-Dorfman syndrome. This study was particularly interesting because it indicated that at least some functions of neutral lipid homeostasis are similar between animals and plants.

The presence of monoacylglycerol (MAG) in plant tissues has been reported previously [7]–[10]. Yang et al. [11] recently demonstrated that the *A. thaliana* GPAT4 and GPAT6 enzymes produce *sn2*-MAG using α,ω-dicarboxylic and ω-hydroxy acyl-CoA as acyl-donors. The MAG product is believed to serve as a precursor for the synthesis of cutin polymers. Also, Reddy et al. described a LysoPA phosphatase in *Arabidopsis thaliana* capable of converting LysoPA to MAG and hypothesized on a possible role in supplying phosphate during low Pi conditions [12]. A similar process has also been reported in yeast [13]. Whilst MAG is therefore not believed to be involved in lipid biosynthesis in plants, this neutral lipid has long been recognised as a central molecule in lipid resynthesis from dietary fat in animals. MAG released by the action of pancreatic lipases is absorbed in the animal intestine and resynthesised into DAG by monoacylglycerol acyltransferase (MGATs) using fatty acyl-CoA as the acyl-donor. *In vitro* studies have demonstrated that this enzyme tends to favour acylation at the *sn*-1 and *sn*-3 position of MAG molecules containing unsaturated moieties at the *sn*-1 or *sn2* position [14]–[16].

The possibility of a MAG or MGAT pathway in plant tissues has been discussed [7]–[10] and one study has demonstrated some soluble MGAT activity in peanut cotyledon extracts [17]. Interestingly, the same group has recently cloned an oleosin protein from the same tissue that displays both MGAT and phospholipase A activities [18]. However, the contribution of this protein to lipid biosynthesis in plant tissues remains to be proven. In this regard the recent characterisation of a soluble DGAT is an interesting addition to the field [19].

Since evidence exists for *de novo* MAG production in plant tissues [11], [12] we explored the feasibility of salvaging this MAG pool as a substrate for TAG synthesis or resynthesis. To this end we expressed a mammalian MGAT in *Nicotiana benthamiana* resulting in a significant increase in the oil content of vegetative tissues. In addition, we provide biochemical evidence for a possible role of GPAT4 in providing endogenous MAG substrate for the heterologous expressed MGAT acyltransferase in vegetative tissues. Based on our findings in both yeast and plant model systems, we propose a novel TAG biosynthesis pathway consisting of only two acyltransferases, GPAT4 and a bifunctional MGAT/DGAT, that are sufficient to convert G-3-P to TAG. By relying on MAG as an intermediate, this alternative pathway could function independently to the endogenous Kennedy pathway in any plant cell and offers potential applications for both food and fuel applications.

## Materials and Methods

### Genes

The *A. thaliana* GPAT4 (Genbank accession NM_100043) was amplified from total RNA isolated from developing siliques. The *Mus musculus* MGAT1 (AAK84177) and MGAT2 (AY157609) genes were synthesised with plant codon usage (Geneart, Regensburg, Germany). The *A. thaliana DGAT1* gene (Genbank accession NM_127503) had previously been amplified from cDNA [17].

### *In vitro* yeast GPAT assay

The GPAT4 and MGAT2 genes were cloned into the dual-promoter yeast expression vector pESC-URA (Agilent Technologies, Santa Clara, CA) either individually or in combination and transformed in the *S. cerevisiae* GPAT knockout strain *gat1*Δ (Matα, his3C1, leu2C0, lys2C0, ura3C0, YKR067w:kanMX4). Yeast transformations were carried out using the Yeast Transformation Kit (Sigma-Aldrich, Castle Hill, NSW Australia). Heterologous expression and preparation of yeast homogenates were essentially as described by [11] except for the use of 0.5 mm zirconium oxide beads and a Bullet Blender (Next Advance, Cambridge, MA) to lyse the cells and resuspension of the final pellet with a Potter-Elvehjen glass homogenizer. GPAT activity was assayed as previously described by Yang et al. [11] except for the use of oleoyl-CoA as the acyl-donor, 0.5 mM G-3-P containing 0.13 µCi [^14^C]G-3-P for 30 minutes in a final reaction volume of 40 µL. Reactions were stopped by adding 6.7 µL acetonitrile/acetic acid (4/1 v/v).

Total lipids were extracted with chloroform/methanol/0.1 M KCl (2/1/1 v/v/v). Reaction mixtures were centrifuged (1500 g for 5 minutes), the lower phase collected and the upper phase extracted a second time with 600 µL chloroform. Total lipid from the combined chloroform phases were separated by two-step TLC. Samples were first run halfway on a pre-coated SIL G-25 TLC plate (Macherey-Nagel, Germany) in chloroform/methanol/acetic acid/water (90/15/10/3 v/v/v/v), followed by a second separation using hexane/diethyl ether/acetic acid (70/30/1 v/v/v). Different lipid fractions were identified by running appropriate standards alongside and staining with iodine vapour. The TLC plate was exposed to phosphor imaging screens overnight and analysed by a Fujifilm FLA-5000 phosphorimager. The radioactivity of each sample was quantified with Fujifilm Multi Gauge software, with several 500 d.p.m. radioactivity spots as reference.

### Transient expression in *N. benthamiana* and lipid analysis

MGAT1, MGAT2 and DGAT1 were expressed in *N. benthamiana* in a transient expression system essentially as described by [20] and [21].

Infiltrated leaf tissue samples were freeze-dried, weighed and total lipids extracted as described by [22]. Equal amounts of lipids and authentic lipid standard spots were applied on a pre-coated silica gel TLC plate (silica gel 60, MERCK) and developed in a solvent system of hexane/diethyl ether/acetic acid (70/30/1 v/v/v) to fractionate neutral lipid and polar lipids classes. Individual bands of lipid classes were visualized by exposing lipids to the iodine vapour and identified according to the migration of lipid standards in the same plate. Fatty acid methyl esters (FAME) of the TAG fractions were produced by collecting the corresponding bands and incubating these in methanol/HCl/dichloromethane (10/1/1 v/v/v) solution for 2 hours at 80°C together with a known amount of heptadecanoic acid (C17:0) as an internal standard to allow quantification. FAME were analysed by GC.

### *N. benthamiana* leaf cell lysate assay and lipid analysis

20,000 g soluble crude microsomal fractions for enzymatic acyltransferase assays were prepared from *N. benthamiana* leaf tissue three days after infiltration. Leaf tissues were ground in a solution containing 0.1 M potassium phosphate buffer (pH 7.2) and 0.33 M sucrose using a glass homogenizer. The leaf homogenate was centrifuged at 20,000 g for 45 minutes at 4°C after which the supernatant was collected. Protein contents of the lysates were measured using a Wallac1420 multilabel counter and a Bio-Rad Protein Assay dye reagent (Bio-Rad Laboratories, Hercules, CA USA). MGAT and DGAT assays were performed according to [23] with some modifications. The reaction medium contained 100 mM Tris-HCl (pH 7.0), 5 mM MgCl_2_, 1 mg/mL fatty acids free-bovine serum albumen (Sigma), 200 mM sucrose, 40 µM cold oleoyl-CoA, 16.4 µM [^14^C]glycerol-labelled *sn2*-monooleoylglycerol (55 mCi/mmol, American Radiochemicals, Saint Louis, MO USA) or 9.0 µM [^14^C]glycerol-3-phosphate disodium salt (50–150 mCi/mmol, American Radiochemicals) and a volume of lysate containing 100 µg protein. The assays were carried out for 7.5, 15 and 30 minutes at room temperature with gentle mixing. Total lipids were extracted as described above but using a chloroform/methanol/0.1 M KCl (2/1/1 v/v/v) solvent run on silica gel 60 TLC plates (MERCK, Dermstadt, Germany) impregnated with 10% boric acid. Plates were developed in hexane/diethyl ether/acetic acid (70/30/1 v/v/v) for TAG and DAG fractionation and developed in chloroform/acetone (90/10 v/v) for MAG before measurement of radiolabel in the lipid spots using a Beckman-Coulter Ready Safe liquid scintillation cocktail and Beckman-Coulter LS 6500 Multipurpose Scintillation Counter.

### Stable expression in *N. benthamiana* and lipid analysis

*N. benthamiana* was transformed essentially as described by [24] for *N. tabacum*. Reverse Transcription (RT)-PCR for expression analysis was performed using Superscript III™ Platinum® One-Step system (Invitrogen, Carlsbad, CA USA) using standard conditions as described in the accompanying literature.

Transgenic T_1_ 35S::MGAT2 events were produced and screened for expression of the MGAT2 transgene by real-time PCR (data not shown). Highly-expressing events were selected and seed produced by these planted out directly onto soil to result in a segregating population of 30 T_2_ seedlings. After three weeks leaf discs were taken from each seedling for DNA extraction and subsequent PCR to determine which lines were transgenic and which were null for the transgene. The population was then harvested with the entire aerial tissue from each seedling cleaned of soil and freeze-dried. The dry weight of each sample was recorded. Total lipids were isolated by addition of 900 µL of chloroform/methanol (2/1 v/v). 3 µg DAGE (diacylglycerol ether, purified by silica column chromatography of shark liver oil using 4% diethyl ether in hexane) was added per 0.3 mg dry leaf weight as internal standard (see below). Samples were homogenized using an IKA ultra-turrax tissue lyser after which 500 µL 0.1 M KCl was added. Samples were vortexed, centrifuged for 5 minutes and the lower phase collected. The remaining upper phase was extracted a second time by adding 600 µL chloroform, vortexing and centrifuging for 5 minutes. The lower phase was recovered and pooled into the previous collection. Lipids were dried under a nitrogen flow and resuspended in 3 µL chloroform per mg leaf dry weight.

One µL of total leaf lipid extract from stably-transformed seedlings was loaded onto Chromarod-SII rods for Iatroscan^TM^ MK-6s TLC-FID analyser (Mitsubishi Chemical Medience Corporation, Japan). The Chromarod rack was then transferred into an equilibrated developing tank containing 70 mL of a Hexane/CHCl_3_/2-Propanol/Formic acid (85/10.7/0.6/0.06 v/v/v/v) solvent system. After 30 minutes of incubation, the Chromarod rack was dried for 3 minutes at 100°C and immediately scanned on the Iatroscan. Peak areas of DAGE internal standard and TAG were integrated using SIC-480II integration software (Version:7.0-E SIC System instruments Co., LTD, Japan). DAGE was used as an internal standard since it does not exist endogenously in *N. benthamiana* extracts. It also showed a good separation from other metabolites and had a similar response factor to *N. benthamiana* TAG.

### HPLC fractionation

HPLC was performed on a Waters system (Milford, MA) consisting of a 600 Controller, 717 plus Autosampler and a 2420 Evaporative Light Scattering Detector (ELSD). Separation of the TAG, DAG and MAG fractions was achieved on a cyanopropyl (CN) polar bonded phase preparative HPLC column (Luna, 250×21.2 mm ID, 5 µm spherical particles, Phenomenex, Torrance, CA) using a solvent gradient of n-hexane or isooctane-methyl tertiary butyl ether (MTBE)-acetone. A 150 µL aliquot of total lipid extract made up in either n-hexane or dichloromethane was loaded onto a 200 µL sample loop. Fractions were collected by means of a flow splitter (Grace Davison Bannockburn, IL) set to a flow split of 96∶4 (collector: detector). The ELSD settings were: data rate 10 point/second, nitrogen pressure 30 PSI, gain 1, time constant 1 second, nebulizer heater level 5% and drift tube temperature 25°C. The regions for collection of TAG, DAG and MAG fractions were determined by analysing a mixture of suitable standards containing 18:0, 18:1^Δ9^, 18:2^Δ9,12^ and 18:3^Δ9,12,15^ fatty acids. Fractions were transmethylated and fatty acids quantified by gas chromatography (GC) using a C19:0 internal injection standard and identifications were confirmed by GC-MS [20].

### Capillary gas-liquid chromatography

FAME were analysed by gas chromatography using an Agilent Technologies 6890N GC (Palo Alto, California, USA) equipped with a 60-m BPX70 column (0.25 mm inner diameter, 0.25 µm film thickness, SGE, Austin, Texas, USA), with the same conditions described previously [25]. Peaks were quantified with Agilent Technologies ChemStation software (Rev B.03-02-SR2 (341)), Palo Alto, California, USA).

## Results

### DGAT activity of MGAT acyltransferases expressed in yeast

The *M. musculus* MGAT2 enzyme had previously been shown to exhibit low DGAT activity [15], [26]. This was confirmed by expressing the mouse MGAT1, MGAT2 and *A. thaliana* DGAT1 acyltransferases in *S. cerevisiae* H1246. This yeast mutant is devoid of DGAT activity and lacks TAG and sterol esters as a result of knockout mutations in four acyltransferase genes (*dga1*, *lro1*, *are1*, *are2*) [27]. *In vivo* feeding with radiolabelled oleic acid substrate revealed that the mouse MGAT1 displays considerable DGAT side-activity (Fig. S1) with 58% of the labelled DAG converted to TAG in contrast with only 1% in the pYES2 control and 71% in the *A. thaliana* DGAT1 assay. Expression of the mouse MGAT2, however, resulted in only small amounts of TAG with only 6% conversion of DAG to TAG. These results are in line with earlier findings obtained with the mouse MGAT1 and human MGAT2 acyltransferases using the Sf-9 expression system [23]. For the remainder of our work we deliberately focused on the *M. musculus* MGAT2 as its low intrinsic DGAT activity simplified interpretation of the assay results and allowed us to better determine contributions of native plant DGATs or other TAG synthesis routes.

### Monoacylglycerol pathway expression in plants

Transient expression of the *A. thaliana* DGAT1 acyltransferase has previously been shown to increase TAG in *N. benthamiana* leaf tissue [20]. We tested the *M. musculus* MGAT2 acyltransferase under the control of the constitutive 35S promoter in the same system both in the presence and absence of the *A. thaliana* DGAT1 acyltransferase. Triplicate infiltrated leaf samples were harvested after three days and partially-purified cell lysates prepared by mechanical tissue lysis followed by centrifugation.

**Figure 2:**
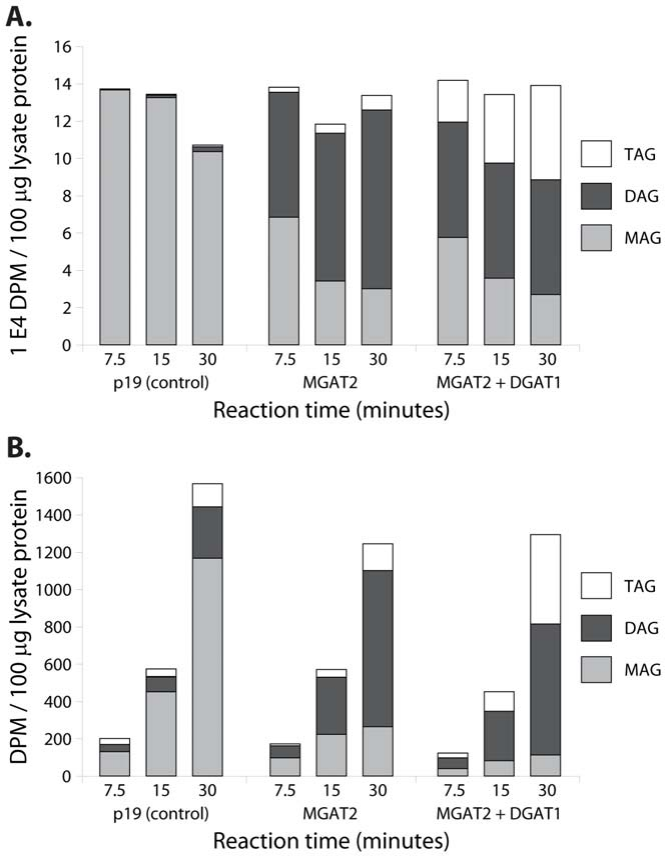
Radioactivity (disintegrations per minute, DPM) of monoacylglycerol (MAG), diacylglycerol (DAG) and triacylglycerol (TAG) fractions isolated from transiently-transformed *N. benthamiana* leaf lysates at three different time points and after feeding with **A.** [^14^C]*sn2*-MAG and unlabelled oleoyl-CoA or **B.** [^14^C] glycerol-3-phosphate and unlabelled oleoyl-CoA. *N. benthamiana* leaves were infiltrated in triplicate with either the 35S::p19 construct alone (negative control), 35S::p19 and 35S::MGAT2 or 35S::p19 and 35S::MGAT2 and 35S::DGAT1 constructs.

The MGAT activities of the cell lysates were assayed by adding labelled *sn2*-MAG and unlabelled oleoyl-CoA to each sample followed by quantification of the labelled DAG and TAG reaction products at three different time points (Fig. 2A). Only traces of labelled DAG and TAG were observed in the p19 control sample at all time points tested. In contrast, expression of the *M. musculus* MGAT2 resulted in 77% of the labelled MAG substrate being converted to DAG and TAG after 30 minutes. DAG to TAG conversion was calculated to be 7% after 30 minutes, likely due to a combination of endogenous DGAT acyltransferases, low intrinsic DGAT activity of the heterologous expressed MGAT2 and other TAG synthesis routes that are active in the plant cell. The amount of label appearing in the DAG and TAG fractions was found to be 80% after 30 minutes when leaf tissue was co-infiltrated with the MGAT2 and *A. thaliana* DGAT1. The DAG produced by the MGAT2 acyltransferase was readily accessible for further conversion to TAG by the co-expressed DGAT1 with 45% being converted after 30 minutes.

Next we confirmed the presence of MAG in wild type *N. benthamiana* leaf tissue by separating total lipids by HPLC followed by GC quantification of the methylated MAG, DAG and TAG fractions in triplicate samples. All three neutral lipid species were present in low concentrations in mature leaf tissue with the amount of fatty acids derived from MAG, DAG and TAG fractions found to be 0.71±0.13 mg, 0.54±0.03 mg and 0.52±0.14 mg per 100 mg dry leaf weight, respectively.

We repeated the above partially-purified cell lysate assay with labelled G-3-P and unlabelled oleoyl-CoA (Fig. 2B) to determine whether this relatively small pool of endogenous MAG was the result of *de novo* synthesis rather than TAG degradation. Labelled MAG accumulated over time in all samples. In the case of the p19 negative control, only 25% of the label was found in DAG and TAG lipid pools after 30 minutes suggesting minimal conversion by the endogenous Kennedy pathway under the conditions of this assay. By contrast, samples infiltrated with MGAT2 were found to have 79% of the label in the DAG and TAG lipid pools after 30 minutes whilst this was increased to 91% for samples co-infiltrated with both MGAT2 and DGAT1. This indicated further conversion of the MAG produced from the labelled G-3-P to DAG and TAG.

### MGAT-mediated TAG accumulation in plants

**Figure 3:**
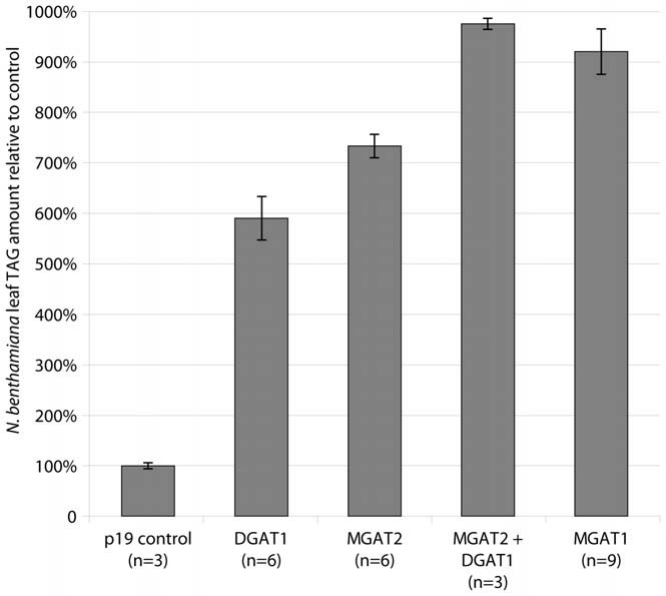
TAG levels in transiently-transformed *N. benthamiana* leaf tissue relative to the p19 negative control with the average amount of TAG in the control samples being 47 µg/100 mg dry weight. Genes expressed include *A. thaliana* DGAT1 (DGAT1); *M. musculus* MGAT2 (MGAT2); *M. musculus* MGAT1 and coexpression of the *M. musculus* MGAT2 and *A. thaliana* DGAT1 (MGAT2+DGAT1). Error bars denote standard error and number of repeats in each case is displayed in parentheses.

The *A. thaliana* DGAT1, *M. musculus* MGAT1, *M. musculus* MGAT2 and a combination of these acyltransferase were expressed transiently in *N. benthamiana* leaf to compare their effect on TAG accumulation. Heterologous expression of MGAT2 and MGAT1 yielded TAG levels that exceeded those of the DGAT1 positive control (7.3-fold, 9.2-fold and 5.9-fold compared to the p19 negative control respectively) (Fig. 3). Co-infiltration with MGAT2 and DGAT1 resulted in the highest TAG yield (9.8-fold compared to p19).

**Figure 4:**
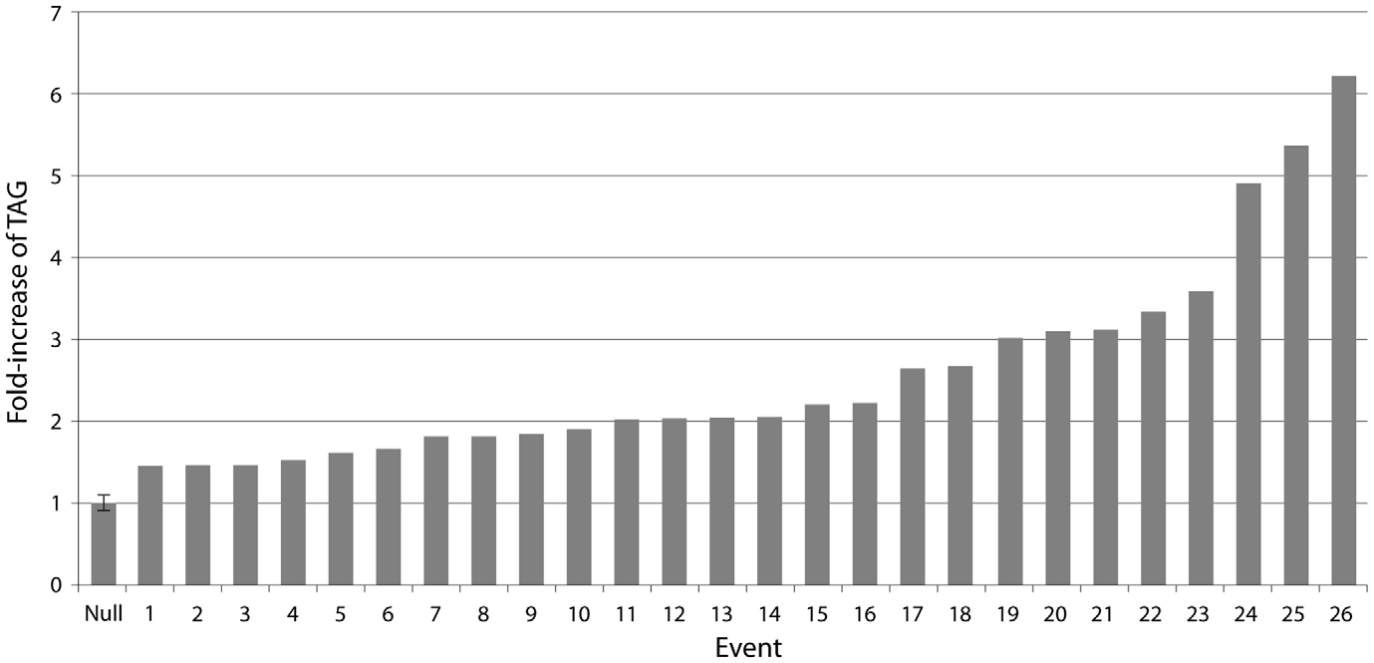
TAG levels in stably-transformed T_2_ segregating *N. benthamiana* aerial seedling tissue. Total lipids were analysed by TLC-FID using an internal DAGE standard to allow accurate comparison between samples. The null column represents the average of eight lines with standard deviation shown.

The 35S::MGAT2 construct was stably-transformed in *N. benthamiana* to rule out any effects on MGAT-mediated TAG increase due to the nature of the transient expression system. The entire aerial tissue from 22 T_2_ segregating seedlings from a highly-expressing and morphologically normal event were analysed for TAG content by TLC-FID. The average TAG level in the transgenic seedlings was found to be 2.6-fold higher compared to eight null seedlings with TAG levels in the highest event increased 6.2-fold (Fig. 4). The median fold increase was found to be 2.0. Mature leaf tissue from the line with the highest TAG was stained with Nile Blue and viewed by confocal microscopy (Fig. S2). Total lipids from leaves of transgenic lines were transmethylated and analysed by GC to determine fatty acid profile. Oleic acid levels were found to be slightly increased compared to control lines and no dicarboxylic or ω-hydroxylated fatty acids [11] were detected in the TAG (Table 1).

**Table 1:** Major fatty acids of total lipids isolated from representative null and transgenic MGAT2 *N. benthamiana* seedlings.

	16:0	16:1^Δ3t^	18:0	18:1^Δ9^	18:1^Δ11^	18:2^Δ9,12^	18:3^Δ9,12,15^
**Null**	15.7	0.3	2.7	4.5	0.4	13.8	61.0
**MGAT2**	16.9	0.4	3.6	6.3	0.5	12.5	58.7

### MAG synthesis by the *A. thaliana* GPAT4 acyltransferase in yeast

**Figure 5:**
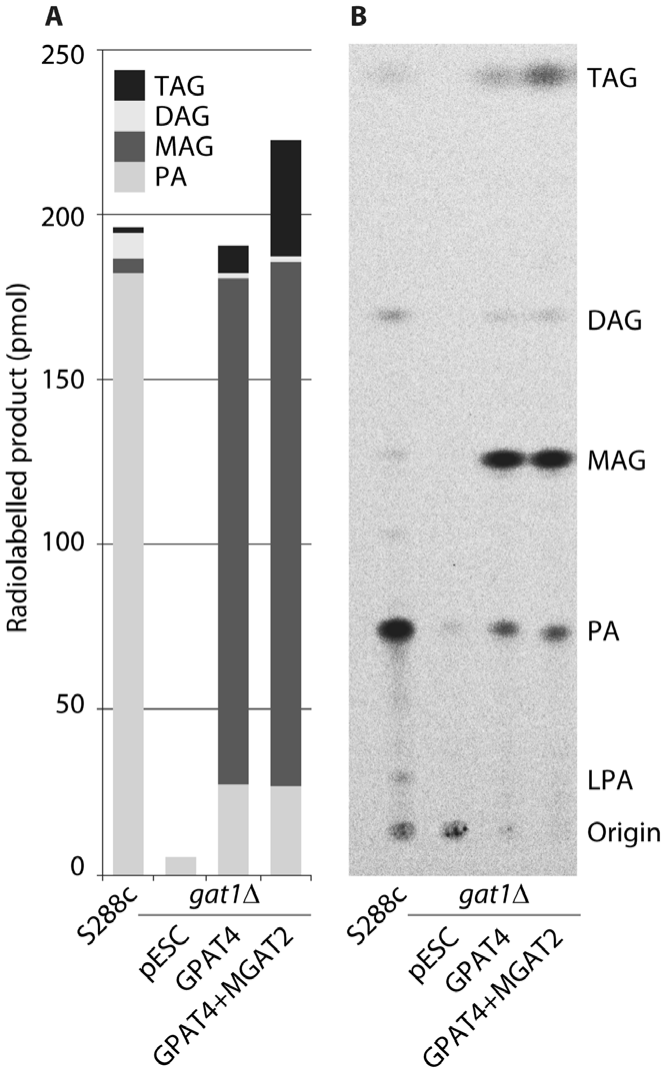
MAG production *in vitro* by the *A. thaliana* GPAT4 acyltransferase expressed in *S. cerevisiae gat1*Δ. Yeast homogenates were incubated for 30 minutes in the presence of labelled glycerol-3-phosphate and oleoyl-CoA after which neutral and polar lipids were separated in a two-step TLC system as described under Materials and Methods. Homogenates prepared from *S. cerevisiae* S288c allowed assessment of the contribution of the endogenous Kennedy pathway whilst the *M. musculus* MGAT2 coexpression tested the alternative MGAT pathway concept. **A.** Quantification of lipid fraction of GPAT assay (numbers in pmoles). 5% of the MAG (8.5 pmole) produced by the GPAT4 transformant was converted to TAG and this increased to 16% (35.3 pmole) with the addition of the MGAT2. **B.** TLC plate showing MAG production by GPAT4.

*A. thaliana* GPAT4 and GPAT6 are highly expressed in vegetative tissues where they synthesise *sn2*-MAG using dicarboxylic and ω-hydroxy acyl-CoA fatty acids [11]. In order to determine whether the *A. thaliana* GPAT4 can catalyse the same reaction using oleoyl-CoA as the acyl-donor, we expressed the gene in the yeast GPAT mutant *gat1*Δ [28] and performed *in vitro* GPAT assays in the presence of labelled G-3-P. Labelled MAG product was present only in trace amounts in homogenates prepared from *S. cerevisiae* S288c after 30 minutes incubation while PA constituted the major labelled lipid species (Fig. 5A and B). When the GPAT4 acyltransferase was expressed in the yeast *gat1*Δ strain, the majority of the label accumulated as MAG. In addition, low levels of labelled DAG and TAG were also detected. Co-expression of the *M. musculus* MGAT2 gene resulted in increased levels of labelled TAG as compared to the expression of GPAT4 gene alone (35.3 pmoles vs. 8.5 pmoles or 16% vs. 5% MAG to TAG conversion).

## Discussion

This study is the first report on the use of MAG as a substrate for *de novo* transgenic TAG biosynthesis in plants. While four different enzymes are involved in the endogenous Kennedy pathway, our proposed MGAT pathway requires only two acyltransferases for the conversion of G-3-P to TAG. The first step consists of the synthesis of *sn2*-MAG by GPAT4 or GPAT6 acyltransferases. Both enzymes have been reported to use dicarboxylic and ω-hydroxy acyl-CoA fatty acids and produce *sn2*-LPA, rather than *sn*-1-LPA. Subsequent dephosphorylation of *sn2*-LPA, catalysed by the same enzyme, results in the production of *sn2*-MAG which serves as a substrate for the MGAT acyltransferase. The last step consists of the acylation of DAG at the *sn-3* position, a reaction catalysed by both DGAT and to some extent by most MGAT acyltransferases. As a result, the concerted action of GPAT4 or GPAT6 and a bifunctional M/DGAT such as the mouse MGAT1 is sufficient to convert the ubiquitous G-3-P to TAG (Fig. 1).

Using a yeast model system, we have demonstrated that the *A. thaliana* GPAT4 can also accept common acyl donors such as oleoyl-CoA, thereby producing MAG. *In vitro* GPAT assays using wild type *S. cerevisiae* homogenates and labelled G-3-P resulted in only minor amounts of labelled MAG, DAG and TAG whilst PA contained the majority of the label. This result reflects the activity of the Kennedy pathway enzymes at the time point studied [29] while MAG might be formed by DAG hydrolysis. However, we cannot rule out that DAG is also the result of MGAT activity of the endogenous DGAT2 (*Dga1*) enzyme which has been shown to account for 60% of the total MGAT activity in yeast cells [30]. In contrast, MAG, DAG or TAG were not detected in a similar assay using homogenates prepared from the *gat1*Δ (GPAT) knockout strain. Expression of the *A. thaliana* GPAT4 in this mutant background predominantly resulted in the production of MAG while DAG and TAG only constituted minor amounts. Coexpression of the MGAT2 acyltransferase further increased TAG yields although conversion rates were low. This is likely a result of the suboptimal conditions of our *in vitro* GPAT assay conditions for the coexpressed MGAT and the endogenous DGAT2 acyltransferases. Although the presence of the endogenous Kennedy pathway acyltransferases complicates interpretation of the fate of MAG produced by the *A. thaliana* GPAT4 *in vitro*, it is tempting to speculate that MAG is further converted to TAG by the coexpressed MGAT2 and endogenous yeast DGAT2 enzymes as we only detected low intrinsic DGAT activity of the MGAT2 acyltransferase when expressed in yeast. Future coexpression experiments in yeast using optimal *in vitro* assay conditions for both GPAT4 and MGAT acyltransferases should allow better following of the flux of label from G-3-P via MAG to TAG.

Proof of concept for MGAT-mediated TAG increase in plant tissues was demonstrated in the model plant *N. benthamiana.* Partially-purified cell lysates from transiently-transformed *N. benthamiana* leaf tissue proved to be a useful and rapid assay system. *In vitro* feeding of leaf cell lysates with labelled *sn2*-MAG and G-3-P demonstrated that leaf tissue can synthesise MAG from G-3-P which can serve as substrate for a heterologous expressed MGAT acyltransferase resulting in increased DAG levels. Further conversion to TAG is likely the results of the endogenous plant DGAT acyltransferase. Although the origin of endogenous MAG in *N. benthamiana* leaves was not established in this study, we believe GPAT4 is a likely candidate based on our *in vitro* yeast assay results and the high expression levels in vegetative tissues. In addition, MGAT2 expression resulted in a net TAG increase which is not expected if MAG solely originates from dephosphorylation of the Kennedy pathway intermediate *sn1*-LPA as reported in *A. thaliana* [12]. Surprisingly, transient expression of MGAT2 in *N. benthamiana* leaves resulted in a greater TAG increase than in the case of the *A. thaliana* DGAT1 expressed in the same system. This was somewhat unexpected since feeding experiments with the partially-purified MGAT2 expressing cell lysates resulted in the accumulation of DAG, rather than TAG, in the absence of heterologous expressed DGAT1. It seems reasonable to assume that DAG produced by the MGAT2 acyltransferase is subsequently converted to TAG by native mechanisms including DGAT1 which may be upregulated in response to the DAG accumulation. However, further investigation is required to confirm this.

DAG plays an important role in lipid metabolism as a precursor of TAG and membrane phospholipids and acts as a secondary messenger in cellular signalling [31]. In mammalian cells many pathways exist that counter build up of DAG, one of these being the low energy-consuming method of converting DAG to storage lipid TAG [31]–[33]. It is therefore not surprising that plant cells have developed several conversion routes for DAG. It is possible that the increased TAG observed in the MGAT2 leaf samples is due to (1) direct conversion of DAG to TAG by endogenous DGAT activity; (2) DAG-DAG transacylase activity resulting in TAG and MAG [34]; or (3) conversion of DAG to phosphatidylcholine (PC) by the CDP-choline∶diacylglycerol cholinephosphotransferase or phosphatidylcholine∶diacylglycerol cholinephosphotransferase after which TAG could be produced either directly by PDAT or by conversion back to DAG [35]–[36]. Bates et al. [2]–[3] demonstrated that, at least in some plants, the majority of DAG produced *de novo* by the Kennedy pathway is not necessarily channelled directly to TAG but rather to PC. It remains to be seen whether this is also the case for DAG produced by a heterologous expressed MGAT acyltransferase or whether the DAG is channelled to TAG. Regardless, we did find that the combination of MGAT2 with DGAT1 resulted in a greater increase in TAG in the transient assay than either the MGAT2 or DGAT1 and that a similar increase was achieved using the bifunctional MGAT1.

An increase in TAG was also observed in *N. benthamiana* seedlings, stably-transformed with the 35S::MGAT2 construct. We used a segregating T_2_ population to provide randomly-distributed null siblings as strong temperature and light effect controls for the experimental design. Whilst the null seedlings displayed a range of TAG levels the transgenic population clearly contained increased levels of TAG. Up to 6.2-fold increase was observed in the best-performing events which is in the same range as previously-described transgenic strategies [5]. The magnitude of TAG increase in both the transiently- and stably-transformed plants was unexpected given the small size of the native leaf MAG pool. The TAG levels we observed exceeded the apparent supply of MAG suggest that the leaf rapidly replenishes the MAG pool while it is being depleted by transgenic MGAT activity. Microscopy demonstrated that at least some of the additional neutral lipids were located within the leaf. It will be interesting to see whether similar accumulation also takes place in other sections of these transgenic plants.

In conclusion, we propose a novel TAG biosynthesis pathway in plants that is partly similar to the well established MGAT pathway in animals. Coexpression of a plant GPAT acyltransferase such as the *A. thaliana* GPAT4 or GPAT6 which can synthesise MAG and a mammalian bifunctional M/DGAT acyltransferase such as the mouse MGAT1 enzyme could result in the biosynthesis of storage lipids while bypassing the Kennedy pathway altogether. Whilst we have demonstrated the feasibility in a plant leaf system, this concept should be applicable to any tissue that contains G-3-P and acyl-CoA substrates. It will be interesting to determine whether a transgenically expressed monoacylglycerol pathway in plants is subject to the same regulation as the endogenous Kennedy pathway and how it might interact with other transgenic strategies that can increase TAG levels. Effects on cutin production also remain to be seen although no significant morphological abnormalities were observed in the stably-transformed lines generated in this study. A transcriptome-based study is being undertaken to determine effects on global gene expression in the MGAT2-expressing transgenic *N. benthamiana* lines.

## Supporting Information

Figure S1 **TAG-formation by DGAT1 but not MGAT2 in yeast assays.** *S. cerevisiae* H1246 [27] was transformed with the pYES2 construct (negative control), *A. thaliana* DGAT1 in pYES2 or *M. musculus* MGAT2 in pYES2 and fed [^14^C]oleic acid. FFA denotes free fatty acid. DGAT activity was assayed by first diluting a preculture to a starting OD_600_ of 0.15 in 5 mL minimal dropout medium lacking uracil and containing 2% galactose, 0.01% NP40 and 1 µCi [14^C^]oleic acid (58 mCi/mmol) (GE Healthcare, Rydalmere, New South Wales, Australia) dissolved in ethanol. Next, cultures were incubated at 28°C with shaking for 3 days and washed 3 times with an equal volume of water before total lipids were isolated.
(TIF)

Figure S2 **Nile Blue lipid staining of** ***N. benthamiana*** **leaf cross-sections viewed under a fluorescence microscope.** **A.** Non-transformed wildtype tissue. **B.** *N. benthamiana* stably-transformed with *M. musculus* MGAT2 driven by the constitutive 35S promoter. Fully expanded young leaves of wildtype and transgenic *N. benthamiana* plants were harvested and immersed in a 1% aqueous solution of Nile Blue A and infiltrated under vacuum for 15 minutes. The samples were then briefly rinsed in distilled water and cross sections of approximately 0.5 mm in thickness were cut under dissection microscope. Neutral lipids were visualized using an UV filter fitted on a Zeiss fluorescence microscope (Carl Zeiss, UK).
(TIF)

## References

[1] Kennedy EP (1961). Biosynthesis of complex lipids.. Fed Proc.

[2] Bates PD, Ohlrogge JB, Pollard M (2007). Incorporation of newly synthesised fatty acids into cytosolic glycerolipids in pea leaves occurs via acyl editing.. J Biol Chem.

[3] Bates PD, Durrett TP, Ohlrogge JB, Pollard M (2009). Analysis of acyl fluxes through multiple pathways of triacylglycerol synthesis in developing soybean embryos.. Plant Physiol.

[4] Weselake RJ, Taylor DC, Rahman MH, Shah S, Laroche A (2009). Increasing the flow of carbon into seed oil.. Biotechnol Adv.

[5] Vega-Sánchez ME, Ronald PC (2010). Genetic and biotechnological approaches for biofuel crop improvement.. Curr Opin Biotech.

[6] James CN, Horn PJ, Case CR, Gidda SK, Zhang D (2010). Disruption of the *Arabidopsis* CGI-58 homologue produces Chanarin–Dorfman-like lipid droplet accumulation in plants.. P Natl Acad Sci USA.

[7] Hirayama O, Hujii K (1965). Glyceride structure and biosynthesis of natural fats III. Biosynthetic process of tryglycerides in maturing soybean seed.. Agr Biol Chem Tokyo.

[8] Panekina T, Gusakova S, Tabak MY, Umarov A (1978). Neutral lipids of the seeds of *Eremostachys moluccelloides*.. Chem Nat Compd.

[9] Lakshminarayana G, Kaimal TNB, Gopalakrishnan N (1984). Changes in lipid class and fatty acid compositions during maturation of Hibiscus esculentus and Hibiscus cannabinus seeds.. J Am Oil Chem Soc.

[10] Perry HJ, Harwood JL (1993). Radiolabelling studies of acyl lipids in developing seeds of *Brassica napus*: use of [1-14^C^] acetate precursor.. Phytochemistry.

[11] Yang W, Pollard M, Li-Beisson Y, Beisson F, Feig M (2010). A distinct type of glycerol-3-phosphate acyltransferase with sn2 preference and phosphatase activity producing 2-monoacylglycerol.. P Natl Acad Sci USA.

[12] Reddy VS, Rao DKV, Rajasekharan R (2010). Functional characterization of lysophosphatidic acid phosphatase from *Arabidopsis thaliana*.. Biochim Biophys Acta.

[13] Reddy VS, Singh AK, Rajasekharan R (2008). The *Saccharomyces cerevisiae PHM8* gene encodes a soluble magnesium-dependent lysophosphatidic acid phosphatise.. J Biol Chem.

[14] Yen CLE, Stone SJ, Cases S, Zhou P, Farese RV (2002). Identification of a gene encoding MGAT1, a monoacylglycerol acyltransferase.. P Natl Acad Sci USA.

[15] Cao J, Lockwood J, Burn P, Shi Y (2003). Cloning and functional characterization of a mouse intestinal acyl-CoA:monoacylglycerol acyltransferase, MGAT2.. J Biol Chem.

[16] Yen CLE, Farese RV (2003). MGAT2, a monoacylglycerol acyltransferase expressed in the small intestine.. Biochemistry-US.

[17] Tumaney AW, Shekar S, Rajasekharan R (2001). Identification, purification, and characterization of monoacylglycerol acyltransferase from developing peanut cotyledons.. J Biol Chem.

[18] Parthibane V, Rajakumari S, Venkateshwari V, Iyappan R, Rajasekharan R (2012). Oleosin is bifunctional enzyme that has both monoacylglycerol acyltransferase and phospholipase activities.. J Biol Chem.

[19] Rani SH, Krishna THA, Saha S, Negi AS, Rajasekharan R (2010). Defective in Cuticular Ridges (DCR) of *Arabidopsis thaliana*, a gene associated with surface cutin formation, encodes a soluble diacylglycerol acyltransferase.. J Biol Chem.

[20] Wood CC, Petrie JR, Shrestha P, Mansour MP, Nichols PD (2009). A leaf-based assay using interchangeable design principles to rapidly assemble multistep recombinant pathways.. Plant Biotechnol J.

[21] Voinnet O, Rivas S, Mestre P, Baulcombe D (2003). An enhanced transient expression system in plants based on suppression of gene silencing by the p19 protein of tomato bushy stunt virus.. Plant J.

[22] Bligh EG, Dyer WJ (1959). A rapid method of total lipid extraction and purification.. Can J Biochem Phys.

[23] Cao J, Cheng L, Shi Y (2007). Catalytic properties of MGAT3, a putative triacylglycerol synthase.. J Lipid Res.

[24] Bouvier-Nave P, Benveniste P, Oelkers P, Sturley SL, Schaller H (2000). Expression in yeast and tobacco of plant cDNAs encoding acyl CoA:diacylglycerol acyltransferase.. Eur J Biochem.

[25] Zhou X-R, Green A, Singh S (2011). *Caenorhabditis elegans* Δ12-desaturase FAT-2 is a bifunctional desaturase able to desaturate a diverse range of fatty acid substrates at the Δ 12 and Δ 15 positions.. J Biol Chem.

[26] Cao J, Burn P, Shi Y (2003). Properties of the mouse intestinal acyl-CoA:monoacylglycerol acyltransferase, MGAT2.. J Biol Chem.

[27] Sandager L, Gustavsson MH, Ståhl U, Dahlqvist A, Wiberg E (2002). Storage lipid synthesis is non-essential in yeast.. J Biol Chem.

[28] Zou J, Zheng Z (2001). The initial step of the glycerolipid pathway.. J Biol Chem.

[29] Athenstaedt K, Daum G (2011). Lipid storage: yeast we can.. Eur J Lipid Sci Technol.

[30] Heier C, Taschler U, Rengachari S, Oberer M, Wolinski H (2010). Identification of Yju3p as functional orthologue of mammalian monoglyceride lipase in the yeast *Saccharomyces cerevisiae*.. Biochim Biophys Acta.

[31] Carrasco S, Merida I (2007). Diacylglycerol, when simplicity becomes complex.. Trends Biochem Sci.

[32] Igal RA, Matías Caviglia J, de Gómez Dumm INT, Coleman RA (2001). Diacylglycerol generated in CHO cell plasma membrane by phospholipase C is used for triacylglycerol synthesis.. J Lipid Res.

[33] Chibalin AV, Leng Y, Vieira E, Krook A, Björnholm M (2008). Downregulation of diacylglycerol kinase delta contributes to hyperglycemia-induced insulin resistance.. Cell.

[34] Stobart K, Mancha M, Lenman M, Dahlqvist A, Stymne S (1997). Triacylglycerols are synthesised and utilized by transacylation reactions in microsomal preparations of developing safflower (*Carthamus tinctorius* L.) seeds.. Planta.

[35] Vogel G, Browse J (1996). Cholinephosphotransferase and diacylglycerol acyltransferase.. Plant Physiol.

[36] Lu C, Xin Z, Ren Z, Miquel M, Browse J (2009). An enzyme regulating triacylglycerol composition is encoded by the ROD1 gene of *Arabidopsis*.. P Natl Acad Sci USA.

