# Correction: Recruiting a New Substrate for Triacylglycerol Synthesis in Plants: The Monoacylglycerol Acyltransferase Pathway

**DOI:** 10.1371/annotation/f0547e8e-5cec-4ae4-8348-07f5aa3b8f34

**Published:** 2012-07-09

**Authors:** James R. Petrie, Thomas Vanhercke, Pushkar Shrestha, Anna El Tahchy, Adam White, Xue-Rong Zhou, Qing Liu, Maged P. Mansour, Peter D. Nichols, Surinder P. Singh

The first three authors, James R. Petrie, Thomas Vanhercke, and Pushkar Shrestha, should be noted as contributing equally to this work. 

