# Supplementary figures and images for: Recruiting a New Substrate for Triacylglycerol Synthesis in Plants: The Monoacylglycerol Acyltransferase Pathway
Source: PLoS One. 2012 Apr 16;7(4):e35214. doi: 10.1371/journal.pone.0035214 (PMC3327653; doi:10.1371/journal.pone.0035214)

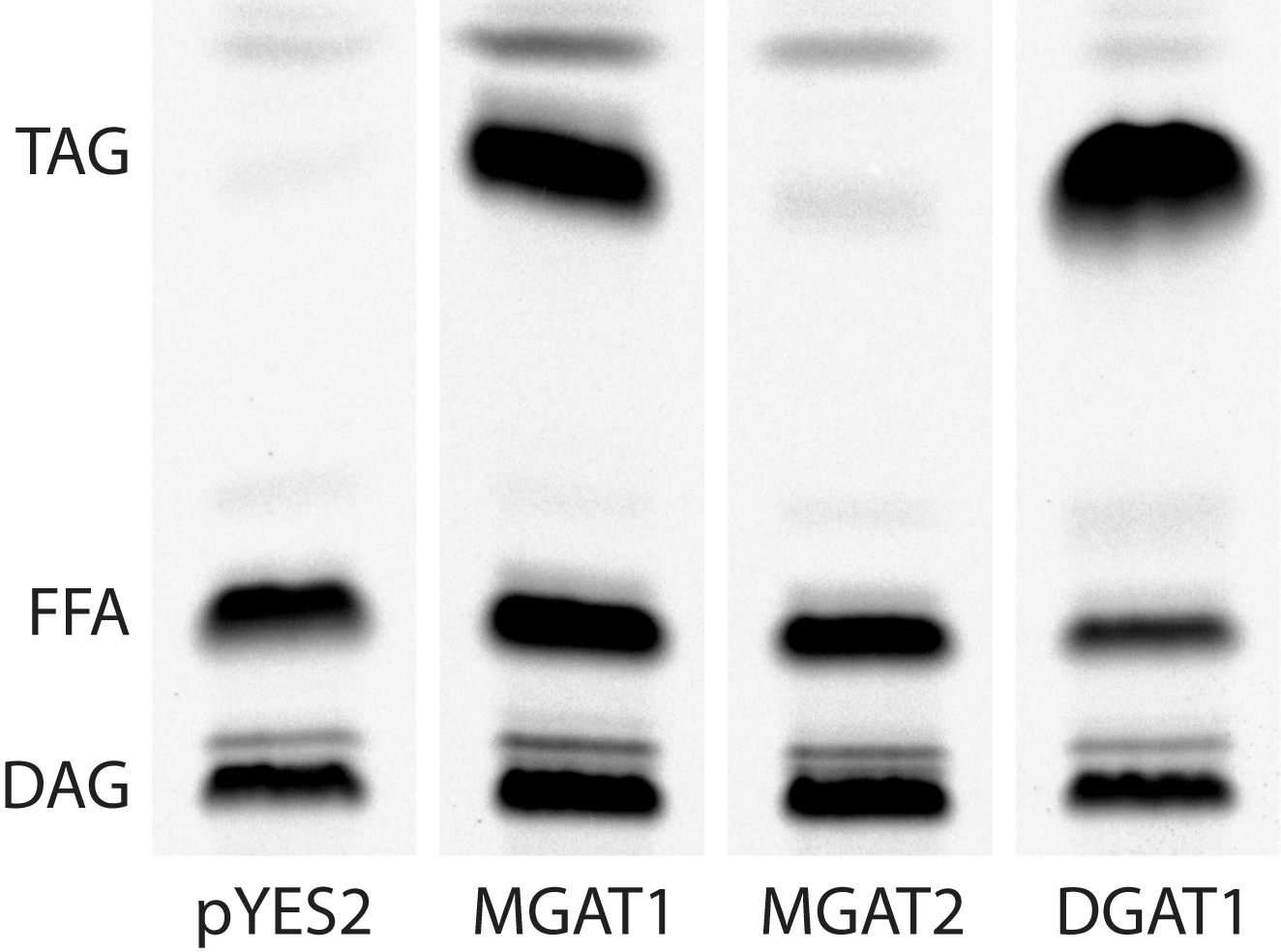

Supplement: Figure S1 — TAG-formation by DGAT1 but not MGAT2 in yeast assays. S. cerevisiae H1246 [27] was transformed with the pYES2 construct (negative control), A. thaliana DGAT1 in pYES2 or M. musculus MGAT2 in pYES2 and fed [14C]oleic acid. FFA denotes free fatty acid. DGAT activity was assayed by first diluting a preculture to a starting OD600 of 0.15 in 5 mL minimal dropout medium lacking uracil and containing 2% galactose, 0.01% NP40 and 1 µCi [14C]oleic acid (58 mCi/mmol) (GE Healthcare, Rydalmere, New South Wales, Australia) dissolved in ethanol. Next, cultures were incubated at 28°C with shaking for 3 days and washed 3 times with an equal volume of water before total lipids were isolated. (TIF) [file pone.0035214.s001.tif]

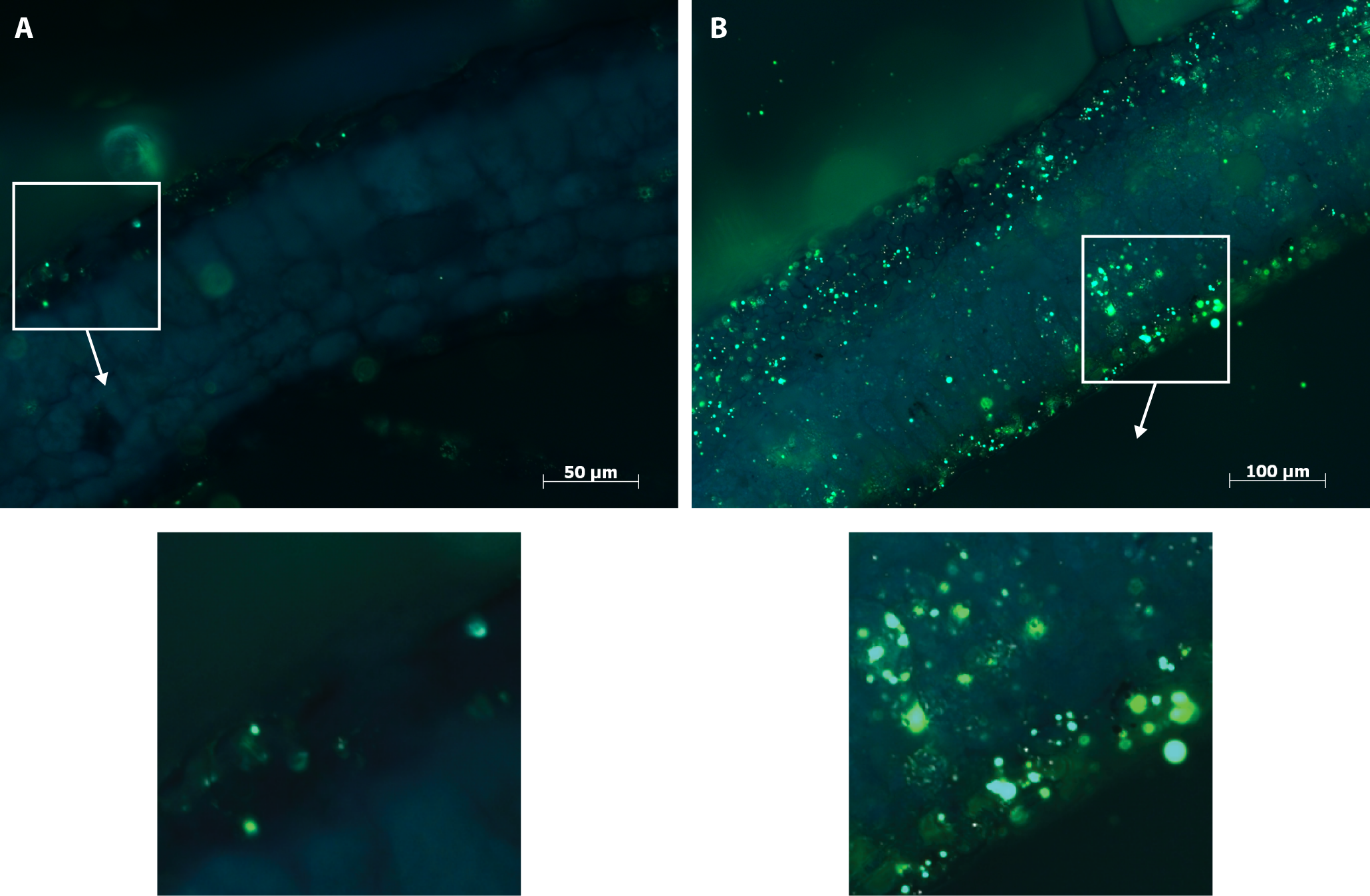

Supplement: Figure S2 — Nile Blue lipid staining of N. benthamiana leaf cross-sections viewed under a fluorescence microscope. A. Non-transformed wildtype tissue. B. N. benthamiana stably-transformed with M. musculus MGAT2 driven by the constitutive 35S promoter. Fully expanded young leaves of wildtype and transgenic N. benthamiana plants were harvested and immersed in a 1% aqueous solution of Nile Blue A and infiltrated under vacuum for 15 minutes. The samples were then briefly rinsed in distilled water and cross sections of approximately 0.5 mm in thickness were cut under dissection microscope. Neutral lipids were visualized using an UV filter fitted on a Zeiss fluorescence microscope (Carl Zeiss, UK). (TIF) [file pone.0035214.s002.tif]
